# Performance Review of Strain-Hardening Cementitious Composites in Structural Applications

**DOI:** 10.3390/ma16155474

**Published:** 2023-08-04

**Authors:** Bingshuang Xue, Binbin Xu, Weihua Lu, Yongxing Zhang

**Affiliations:** 1 School of Civil Engineering, Nanjing Forestry University, Nanjing 210037, China; 2 No.3 Engineering Company Ltd. of CCCC First Harbor Engineering Company, Dalian 116011, China

**Keywords:** review, structural performance, strain-hardening cementitious composites, SHCC member, SHCC strengthening layer

## Abstract

Strain-hardening cementitious composites (SHCC) are an attractive construction material with obvious advantages of large strain capacity and high strength, as well as excellent workability and easy processing using conventional equipment. Moreover, SHCC can be designed with varied mix proportions in order to satisfy various requirements and expectations to overcome the shortages of existing construction materials. However, the behavior of SHCC in the structural application is varied from that of SHCC material, which is reviewed and presented in this paper, focusing on the flexural and shear behavior of the SHCC member and the SHCC layer used for strengthening reinforced concrete (RC). The reviewed results demonstrate that both the zero-span tensile behavior of the stress concentration and the uniaxial tensile behavior of the bending effect can influence the crack propagation patterns of multiple fine cracks in the SHCC strengthening layer, in which the crack distribution within the SHCC layer is limited near the existing crack in the RC substrate member in the zero-span tensile behavior. Moreover, the crack propagation patterns of the SHCC strengthening layer are changed with varied layer thicknesses, and the SHCC strengthening layer, even with a small thickness, can significantly increase the shear load carrying capacity of the shear strengthened RC member. This work provides the foundations for promoting SHCC material in the structural application of repairing or retrofitting concrete structures.

## 1. Introduction

Concrete structures are easily damaged due to the weakness of concrete in resisting tensile forces with brittleness, and many different construction materials have been developed for repairing or strengthening aged and damaged concrete structures. However, the typical existing construction materials have obvious shortages in strengthening concrete structures, in which the performance of the fiber-reinforced polymer (FRP) strengthening layer is strictly related to the bond and compatibility between FRP and concrete [1,2,3]; epoxy-bonded concrete is easily delaminated due to epoxy expansion caused by solar radiation [4], fiber-reinforced concrete (FRC) is still quasi-brittle during the repairing works [5,6,7], and polymer cement mortars (PCM) have limitations in terms of high costs [8].

In recent decades, strain-hardening cementitious composites (SHCC) have been developed with the obvious advantages of a large strain capacity, high strength, self-healing capacity, excellent workability, and easy processing using conventional equipment [9,10,11]. In particular, SHCC can be designed with varied mix proportions, which is expected to overcome the shortages of existing construction materials and satisfy the various requirement in engineering construction. In view of previous studies, the behavior of SHCC in structural applications has varied from that of SHCC materials, whereas the property of SHCC materials is closely connected with the behavior of SHCC in structural applications [12,13,14,15]. Table 1 demonstrates the findings of the reviewed sources on the property of SHCC material and the performance of SHCC in structural applications. Environmental hazards such as temperature and humidity variations can influence the durability of SHCC in structural applications [13]. The self-healing capacity of SHCC material can reduce or even eliminate the maintenance requirement of the structure using SHCC [16]. The large strain capacity of SHCC material with multiple fine cracks can improve the durability of the structure using SHCC [17,18]. The high strength and ductility of SHCC material can enhance the fatigue performance of the SHCC layer, strengthening concrete beams and improving the impact and static behaviors of the thin SHCC layer, strengthening reinforced concrete (RC) slabs [19,20]. Due to the aforementioned advantages, SHCC are suitable for repairing damaged concrete structures or strengthening aged concrete structures in operation, which can have a bright future.

As mentioned above, the behavior of SHCC in structural applications is quite different from that of SHCC material, since the short fibers distributed in large-sized structures using SHCC are significantly varied from those distributed in small specimens for obtaining the material properties of SHCC [22,23]. In this paper, the structural performance of SHCC is systematically reviewed, focusing on the flexural and shear behavior of the SHCC member and the SHCC layer used for strengthening the RC member.

## 2. Material Property of SHCC Material

**Figure 1:**
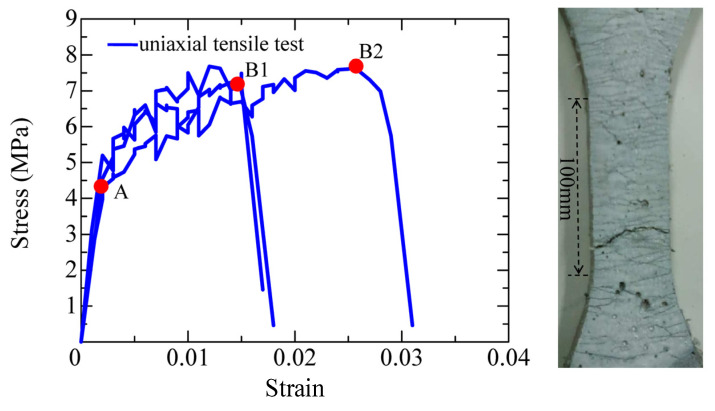
Results obtained from the uniaxial tensile test.

As mentioned above, SHCC can be designed with varied mix proportions, the main ingredients being water, cement, silica fume, fine sand, and fiber. Figure 1 shows the stress-strain curves and cracking pattern of the dumbbell-shaped SHCC specimens, which are obtained from the uniaxial tensile test. The dumbbell-shaped specimens have a 13 mm × 30 mm cross-section, and the strain is defined as the elongation rate with a 100 mm measured length [24]. In the mix proportion of this SHCC material, the water-to-binder ratio is 22%, and there is 338.5 kg of water, 1267.9 kg of cement, 230.8 kg of silica fume, 153.9 kg of fine sand, 14.6 kg of PE fiber, 15.4 kg of super plasticizer, and 0.06 kg of air reducing agent in per cubic meter of the SHCC material. Moreover, this SHCC material is also employed in the following experiment of the SHCC member and the SHCC layer used for strengthening the RC member.

As demonstrated in Figure 1, the initial tension strength of the SHCC material is 4.2 MPa at point A, and the ultimate tensile strengths with a lower limit and an upper limit, is 7 MPa and 8 MPa at points B1 and B2, respectively, corresponding to the hardening strains of 1.5% and 2.2%. In all of the specimens, the initial cracks firstly appear at the initial tension strength at point A, and then the multiple fine cracks with significant strain hardening behavior occur and propagate until the ultimate tension strength at points B1 or B2. Thereafter, the stress decreases due to the localization of the multi-fine cracks.

## 3. Flexural and Shear Behavior of the SHCC Member

### 3.1. Flexural Behavior of the SHCC Member

**Figure 2:**
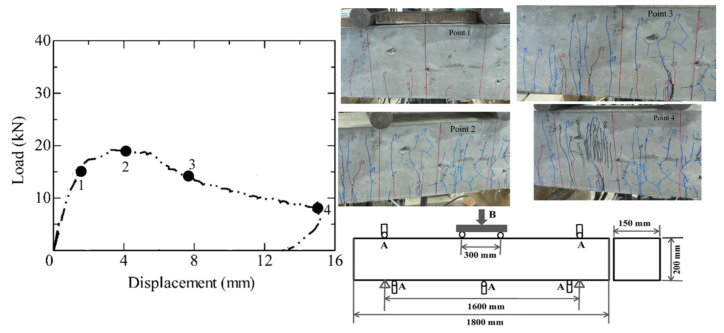
Flexural behavior of the SHCC member.

Figure 2 shows the load-displacement curve and crack propagation pattern of the SHCC member in flexural failure, in which the geometry of the experimental specimen is 1800 mm long with a cross-section of 150 mm × 200 mm. Moreover, there are no stirrups and longitudinal reinforcements arranged in the SHCC member. The specimen is loaded under a four-point bending setup, the displacements and load are measured using displacement transducers and a load cell, respectively, which are marked as A and B, respectively. The range of the load cell is 300 kN, with 0.1 kN sensitivity, and that of the displacement transducer is 25 mm with 0.002 mm sensitivity. During the experiment, the displacement transducer is required to be adjusted when the displacement is larger than 25 mm.

The crack propagation pattern of the SHCC member corresponding to the points 1 to 4 in the load-displacement curve is also shown in Figure 2. It can be clearly seen that the flexural multiple fine cracks appear in the SHCC member first (point 1), and then those are significantly increased with the increasing load carrying capacity (point 2), and finally, fails due to the localization of some flexural multiple fine cracks thereafter (points 3 and 4). This means the SHCC member experiences the multi-cracking processes, whereas the multiple fine cracks distributed in the flexural-failed SHCC member are reduced from those of the SHCC material in uniaxial tension.

### 3.2. Shear Behavior of the SHCC Member

#### 3.2.1. Shear Stress-Displacement Relationship and Crack Propagation Pattern

**Figure 3:**
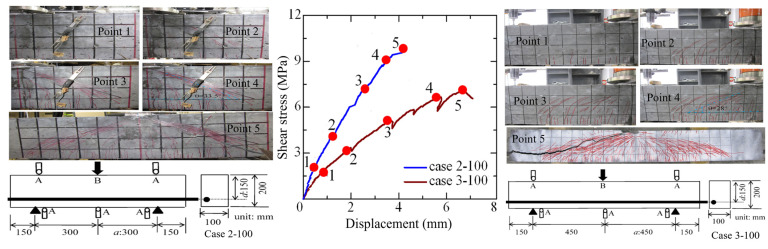
Shear behavior of the SHCC member.

Figure 3 shows the shear stress-displacement curve and crack propagation pattern of the SHCC members in diagonal shear failure [24], in which cases 2-100 and 3-100 have the ratios of shear span length to effective depth (a/d) equaling 2 and 3, respectively. In both cases, 2-100 and 3-100 have a 100 mm width and a 150 mm effective depth, and the shear span lengths of the cases 2-100 and 3-100 are 300 mm and 450 mm, respectively. Moreover, the 25-mm diameter high tensile strength deformed bar with a 200 GPa Young’s modulus and a 1050 MPa yield strength is arranged in the longitudinal direction. In the experiment, the displacements at the loading points and supporting points, as well as the middle point, are measured by displacement transducers, and the load is measured by a load cell, which are marked as A and B, respectively. The loading test is terminated when a sudden drop is observed during the loading process.

The shear stress is defined by the shear force divided by the effective section area of the effective height multiplied by width, and the maximum shear stresses of cases 2-100 and 3-100 are 9.8 MPa and 7.0 MPa, respectively, which means the specimen with a/d = 2 has a larger load carrying capacity than that of the specimens with a/d = 3. The shear stress-displacement curve also shows that the linear curve is near the maximum shear strength and the sudden shear failure occurs thereafter. This behavior may be due to the smooth crack surface of the SHCC member.

The crack propagation pattern of the tested specimens corresponding to the points 1 to 5 in the shear stress-displacement curve is also demonstrated in Figure 3. In the case 2-100, the flexural multi-fine cracks first occur at point 1 (lower load level with a 2 MPa shear stress), and some multiple fine cracks occur in the middle of the shear span independent of the aforementioned flexural cracks at point 2 (load level with 4 MPa shear stress). At points 3 and 4 (load level with 7.3 MPa and 9.3 MPa shear stresses), the multiple fine cracks are gradually increased with the increasing load level, in which the multiple fine cracks in the shear direction play a dominant role in the SHCC member in shear failure. Moreover, it is obvious that the localization behavior occurs among the multiple fine cracks at point 5 (peak load with a 9.8 MPa shear stress), and the shear load suddenly drops thereafter, in which the shear angle of the multiple fine cracks between the diagonal shear direction and axial direction is about 33.5°.

In the crack propagation pattern of case 3-100, it can be easily seen that this specimen shows similar crack propagation behavior to that of case 2-100. The flexural multi-fine cracks first occur at point 1 (a lower load level with a 1.7 MPa shear stress), then the multiple fine cracks occur in the middle of the shear span, in which the multiple fine cracks in the shear direction are increased and play a dominant role in increasing the shear stress from 3.3 MPa to 6.7 MPa (point 2 to 4). Thereafter, the localization of some multiple fine cracks occurs until the peak load with a 7.0 MPa shear stress (point 5), and the specimen is fractured in shear failure, in which the shear angle of the multiple fine cracks between the diagonal shear direction and the axial direction is about 28°.

#### 3.2.2. Characteristics of the SHCC Fracture Surface

**Figure 4:**
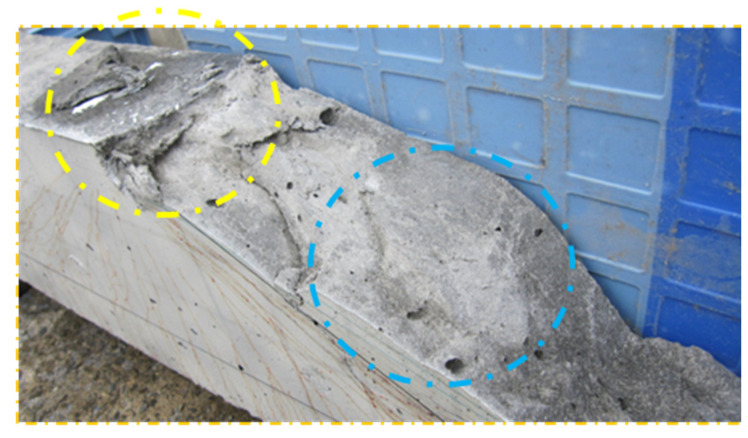
Fracture surface of the SHCC member and normal concrete.

Figure 4 shows the fracture surface of the SHCC member in shear failure. It can be seen that the area near the loading point (yellow line area) is collapsed and the rest of the area (blue line area) is very smooth, whereas the fracture surface of normal concrete is quite rough. Hence, the fracture surface of the SHCC member is different from that of normal concrete. In order to understand the shear failure of SHCC, the fracture surface of the shear-failed SHCC member is surveyed.

**Figure 5:**
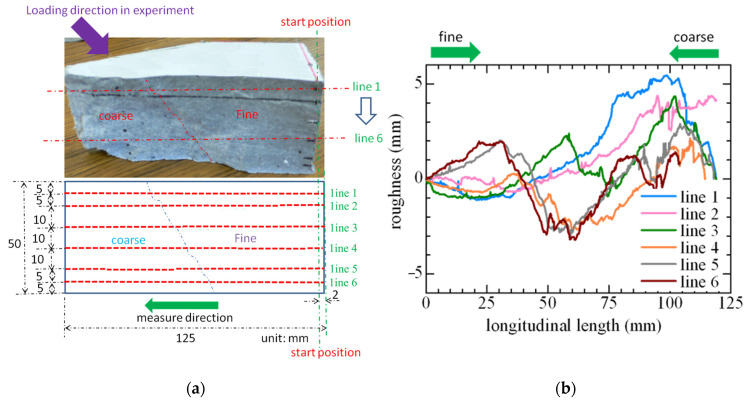
Roughness of the fracture surface in the shear-failed SHCC member. (**a**) Fracture surface. (**b**) Roughness.

Figure 5 demonstrates the investigated roughness of the fracture surface in the shear-failed SHCC member, in which the sample is obtained from the coarse area near the loading point of the tested specimens. The sample length in the longitudinal direction is 125 mm, and the measured length is 123 mm, since the starting position for measurement has a distance of 2 mm from the side. The sample surface is measured using a laser displacement meter, which is implemented along the six dotted red lines, as marked in Figure 5, and six series of roughness (line 1 to line 6) along the dotted red lines are obtained.

Figure 5 shows the revised test data of the measured surface roughness, in which the start position (0 mm position) in the fine area and the end position (125 mm position) in the coarse area have the same roughness change of 0 mm. It can be easily seen that the roughness change of the measured surface is serrated, with many peak-down phenomena, and the roughness in the coarse area is obviously larger and steeper than that in the fine area.

## 4. Flexural and Shear Behavior of the SHCC Layer Strengthening the RC Member

### 4.1. Flexural Behavior of the RC Member with Strengthening Using the SHCC Layer

**Figure 6:**
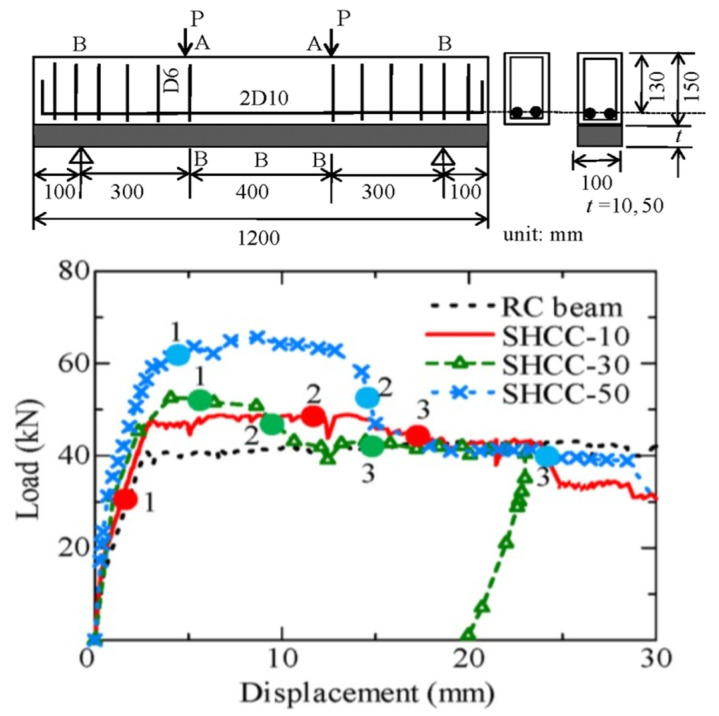
Load vs. displacement of flexural strengthened RC member using the SHCC layer.

Figure 6 shows the load-displacement curves of the SHCC layer strengthening the RC member in flexural failure, in which the tested specimens are loaded under a four-point bending setup. In the experiment, the RC beam is the control beam, and the cases SHCC-10, SHCC-30, and SHCC-50 represent RC beams strengthened by the SHCC layer with different thicknesses of 10 mm, 30 mm, and 50 mm, respectively [25]. The load is measured by a load cell, and the displacements at loading points and supporting points, as well as a middle point, are measured by displacement transducers, which are marked as A and B, respectively.

#### 4.1.1. Load-Carrying Capacities of Strengthened RC Member in Flexural Failure

As shown in Figure 6, it can be clearly seen that the initial stiffness of the load-displacement curve of the strengthened RC member is increased with the increasing thickness of the SHCC layer. The load carrying capacity of the control beam is 40.8 kN, and those of the cases SHCC-10, SHCC-30, and SHCC-50 are 48.9 kN, 55 kN, and 65.7 kN, respectively, which increase by 16.5%, 34.8%, and 61.0% from that of the control beam. Moreover, the cases SHCC-10, SHCC-30, and SHCC-50 exhibit similar characteristics of load-carrying capacity. It is obvious that the initial stiffness of the strengthened RC member first changes after the yielding of the longitudinal reinforcements, and then the SHCC layer continues to carry the load with ductile behavior due to the multiple fine cracks in the SHCC layer. Thereafter, the load drops due to the lost load-carrying capacity of the SHCC layer, which is induced by localized multiple fine cracks in the SHCC layer, and then the load continues to drop due to the gradual failure in the RC substrate beam.

#### 4.1.2. Crack Propagation Pattern of the SHCC Layer for Flexural Strengthening

**Figure 7:**
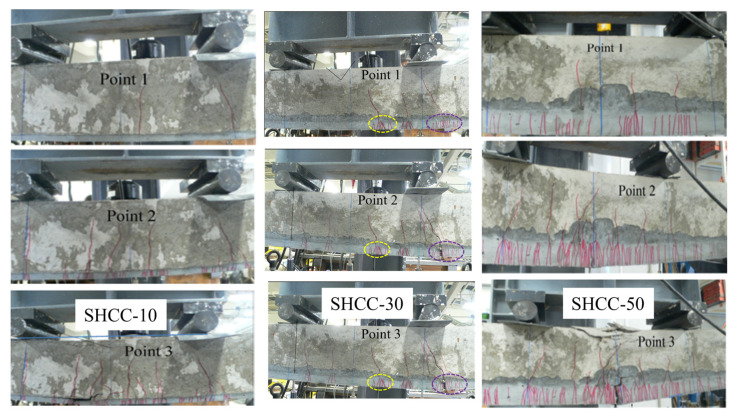
Crack patterns of the RC member with flexural strengthening using the SHCC layer.

The crack propagation pattern of the SHCC layer strengthening the RC member in flexural failure is demonstrated in Figure 7, which corresponds to the points 1 to 3 in the load-displacement curves in Figure 6. In the case of SHCC-10, two localized cracks first occur in the RC substrate beam without multiple fine cracks in the SHCC layer (point 1). After that, the zero-span tensile behavior is dominant in the SHCC layer adjacent to every localized crack in the RC substrate beam (point 2), in which multiple fine cracks occur due to the damage in the SHCC layer caused by the stress concentration from the localized crack in the RC beam. Specifically, those multiple fine cracks from the zero-span tensile behavior are around the localized crack in the RC beam, which spread from the top to the bottom of the SHCC layer. Thereafter, some multiple fine cracks localize in the SHCC layer, which gradually lessens the load-carrying capacity (point 3), and the debonding behavior occurs between the SHCC layer and the RC beam. In the case of SHCC-30, two types of multiple fine cracks first distribute in the SHCC layer (point 1). One type comes from the aforementioned zero-span tensile behavior with many multiple fine cracks around the localized crack in the RC beam, and another type comes from the uniaxial tensile behavior, with many multiple fine cracks spread from the bottom to the top of the SHCC layer. Specifically, those multiple fine cracks from the uniaxial tensile behavior occur due to the bending effect of the strengthened RC beam. Moreover, the SHCC strengthening layer loses its load-carrying capacity due to completing the localization of the multiple fine cracks thereafter, in which the SHCC layer cannot constrain the localized crack in the RC beam to open, and debonding behavior occurs at last. In the case of SHCC-50, the multiple fine cracks in the SHCC layer are almost evenly distributed from the aforementioned uniaxial tensile behavior (point 1), which is increased and quite different from cases SHCC-10 and SHCC-30 (point 2). This may imply that the uniaxial tensile behavior comes from the bending effect that plays a dominant role in the crack propagation pattern in the case of SHCC-50, in which the multiple fine cracks spread from the bottom to the top of the SHCC layer.

**Figure 8:**
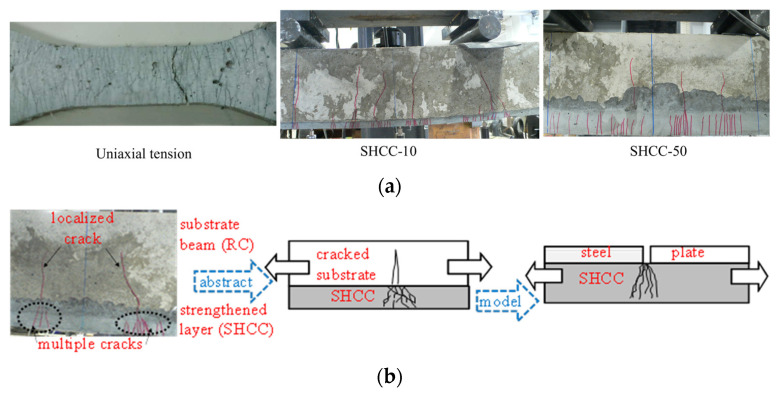
Cracking behavior of the SHCC layer used for RC flexural strengthening. (**a**) Crack patterns of the SHCC material and the SHCC layer used for flexural strengthening. (**b**) Concept of the zero-span tensile test.

Figure 8 demonstrates the crack patterns of the SHCC layer used for the flexural strengthening of the RC member with varied thicknesses of the SHCC layer, compared with the crack pattern of SHCC in uniaxial tension, as shown in the aforementioned Figure 1. It can be clearly seen that the multiple fine cracks are evenly distributed in the uniaxial tensile behavior, whereas those of the SHCC strengthening layer are differently distributed. On one hand, the multiple fine cracks in the SHCC strengthening layer are limited, adjacent to the localized crack in the RC member, while the thickness of the SHCC strengthening layer is small. On the other hand, most of the multiple fine cracks are evenly distributed, while the thickness of the SHCC layer is large, except for the concentrated multiple fine cracks around the localized crack in the RC member. As a result, the obvious advantage of the high ductility of the SHCC material in uniaxial tension is reduced by the effect of the existing crack within the RC substrate member in repair or strengthening applications. Moreover, the zero-span tensile test is recommended to assess the crack opening performance of the thin-thickness SHCC layer used for RC flexural strengthening, in which the crack distribution within the SHCC layer is limited near the existing crack in the RC structure [25,26,27]. As shown in Figure 8, the zero-span tensile test is implemented using the zero span formed by a pair of steel plates, which is modeled considering the condition near the crack in the RC substrate member.

### 4.2. Behavior of the RC Member with Shear Strengthening Using the SHCC Layer

**Figure 9:**
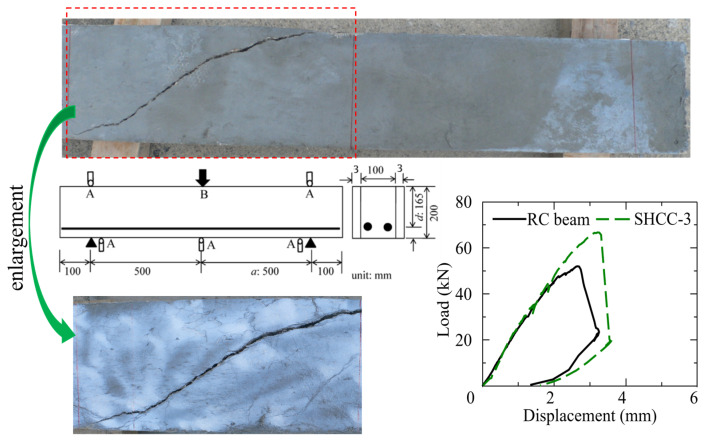
Behavior of the RC member with shear strengthening using the SHCC layer.

Figure 9 shows the load-displacement curves and the crack distributions of the RC member with shear strengthening using the SHCC layer, in which the ratios of the shear span length to the effective depth of all the specimens are 3. The RC beam is the control beam [28], and the case SHCC-3 represents the RC beam strengthened by a 3-mm thick SHCC layer on both sides. Both the RC beam and SHCC-3 have a 1200-mm length and a 100 mm × 200 mm cross-section, in which there are no web reinforcements, and two 10-mm-diameter deformed bars are arranged along the longitudinal direction. In the case of SHCC-3, both side surfaces of the RC beams are roughed by washing out using a retarder, which are adopted as the interfaces of the RC member and the SHCC strengthening layers; the SHCC layers are cast at both sides of the RC beam thereafter. In the experiment, the specimens are loaded under a four-point bending setup; the displacements at the loading points and the support point, as well as the middle point, are measured by displacement transducers, and the load is measured by a load cell, which are marked as A and B, respectively.

As shown in Figure 9, it can be clearly seen that the shear load-carrying capacity of the shear-strengthened RC member is significantly increased, even with the small thickness of the SHCC strengthening layer. This excellent strengthening effect probably originates from the shear stress transfer behavior on the localized crack surface of the SHCC strengthening layer, which is contributed to by both the contact stress and fiber bridging stress on the crack surface of the SHCC layer.

Moreover, it can be seen that the observed multiple fine cracks in the diagonal shear direction of the SHCC strengthening layer are obviously decreased from those of the aforementioned shear-failed SHCC member, as demonstrated in Figure 3. This implies that the aforementioned zero-span tensile behavior is also observed in the SHCC layer used for shear strengthening the RC member, in which the crack distribution within the SHCC layer is limited near the existing crack in the RC member. Therefore, the ductility of SHCC is also reduced while it is used for the shear-strengthening RC member, which is similar to that of SHCC used for the flexural-strengthening RC member.

## 5. Conclusions

In this paper, the structural performance of SHCC is reviewed, focusing on the flexural and shear behavior of the SHCC member and the SHCC layer used for strengthening the RC member; the following conclusions can be drawn.

(1) The shear-failed SHCC members are suddenly dropped and smoothly decreased after reaching the peak load capacity, in which the shear-failed SHCC members demonstrate similar crack propagation behavior. The flexural multi-fine cracks first occur, and then some multiple fine cracks occur in the middle of the shear span independent of the aforementioned flexural cracks, in which the multiple fine cracks are gradually increased with the increasing load level thereafter. The multiple fine cracks in the shear direction play a dominant role in the SHCC member during shear failure. After that, the localization behavior occurs among the multiple fine cracks and the sudden drop in shear load appears in the shear-failed SHCC member.
(2) The crack propagation patterns of the SHCC member in flexural failure demonstrate that the flexural multiple fine cracks appear in the SHCC member first, and then the multiple fine cracks significantly increase with the increasing load-carrying capacity. This means that the SHCC member experiences multi-cracking processes, which finally fail due to the localization of some flexural multiple fine cracks in the SHCC member thereafter.
(3) In the load-displacement curves of the SHCC layer flexural strengthening the RC member, the initial stiffness of the strengthened RC member first changes after yielding the longitudinal reinforcements, and then the SHCC layer carries the load with ductile behavior due to the multiple fine cracks in the SHCC layer. Thereafter, the load drops due to the decreased load-carrying capacity of the SHCC layer, which is induced by the localized multiple fine cracks in the SHCC layer, and then the load continues to drop due to the gradual failure in the RC substrate beam. There are two types of crack propagation patterns in the multiple fine cracks in the SHCC layer used for the flexural strengthening of the RC member, which is influenced by the varied thicknesses of the SHCC layer. The multiple fine cracks from the zero-span tensile behavior are around the localized crack in the RC beam and spread from the top to the bottom of the SHCC layer, which are caused by the stress concentration from the localized crack in the RC beam. The multiple fine cracks from the uniaxial tensile behavior are almost evenly distributed and spread from the bottom to the top of the SHCC layer, which is induced by the bending effect of the strengthened RC beam.
(4) In the SHCC layer used for the shear strengthening of the RC member, the SHCC layer can significantly increase the shear load-carrying capacity of the strengthened RC member. This excellent strengthening effect is probably due to the shear stress transfer behavior on the localized crack surface of the SHCC strengthening layer, which is contributed to by both the contact stress and fiber bridging stress on the crack surface of the SHCC layer. Moreover, the multiple fine cracks in the diagonal shear direction of the SHCC strengthening layer are obviously decreased from those of the shear-failed SHCC member, which means that the ductility of the SHCC layer is reduced while it is used for shear strengthening.

## Figures and Tables

**Table 1:** Findings of reviewed sources on material property and structural application of SHCC.

Authors	Origin	Purpose	Summary Point
Chen et al. (2019) [9]	China	To evaluate the dynamic compressive properties of SHCC	Nanomaterials can improve the strength and toughness of SHCC and nanomaterials-modified SHCC are more sensitive to strain rate
Li and Yang (2018) [10]	Singapore	To assess tensile strain-hardening potential of FRCC	Probabilistic-based model can evaluate uncertainty in tensile strain-hardening potential of FRCC with heterogeneity by treating micromechanical parameters as random variables
Zhang et al. (2009) [11]	China	To assess mechanical performance of ECC with low drying shrinkage	Significant plasticity of ECC is found under compressive load after peak stress, except for similar behavior under tensile load
Li et al. (2018) [12]	USA	To novelly use a super ductile FRCC for repairing concrete structures	The material ductility of the super ductile FRCC can translate into strong and ductile structural performance
Bartosz et al. (2011) [13]	Brazil	To evaluate the durability of SHCC exposed to natural weathering	Temperature and humidity variations can influence the durability of SHCC in structural application
Kim et al. (2004) [14]	Korea	To investigate fundamental performances of sprayed ECC in repair systems	ECC can be effective in extending the service life of rehabilitated infrastructures
Zhang et al. (2015) [15]	Japan	To avoid over congestion at joint connection of rigid-famed railway bridge	PP-ECC is effective in replacing transverse reinforcements in the beam–column joints of railway rigid-framed bridge
Qian et al. (2009) [16]	Netherlands	To study self-healing behavior of pre-cracked fiber reinforced SHCC	The developed self-healing SHCC can potentially reduce or even eliminate the maintenance needs
Wang et al. (2020) [17]	China	To study the influence of interface roughness and repair layer thickness on bonding and shrinkage properties of SHCC-repaired concrete beams	Roughed bonding surface can improve bond strength of SHCC and old concrete, and cracking and delamination of SHCC repair layer are alleviated with increasing SHCC layer thickness
Leung and Cao (2010) [18]	China	To develop pseudo-ductile permanent formwork for durable concrete structures	Permanent formworks can be fabricated as effective surface cover to prevent reinforcement corrosion, using PDCC with relatively low water/binder ratio
Leung and Zhang (2007) [19]	China	To strategically apply ECC in parts of a structure that is under relatively high stress and strain	Applying ECC layer on tensile side of flexural beam can increase flexural strength, ductility, and fatigue life of the beam
Mahmoud et al. (2020) [20]	Egypt	To improve impact resistance of reinforced concrete slabs	Thin SHCC layer added at either tension or compression side of reinforced concrete slab can improve the impact resistance of the slab
Figueiredo et al. (2020) [21]	Netherlands	To evaluate mechanical behavior of printed SHCC	Printing technique guarantees an enhanced bond between the printed SHCC layers

## Data Availability

The data used to support the findings of this study are available from the corresponding author upon request.
